# *Behavioral Sciences*: An International, Open-Access, Peer Reviewed Journal

**DOI:** 10.3390/behavsci1010001

**Published:** 2011-02-22

**Authors:** John Coverdale

**Affiliations:** *Founding Editor-in-Chief of Behavioral Sciences*, Menninger Department of Psychiatry and Center for Medical Ethics, Baylor College of Medicine, Houston, Texas 77030, USA; E-Mail: jhc@bcm.edu

## Abstract

On behalf of the Editorial Board and the editorial management staff of MDPI, it is my great pleasure to introduce this new journal *Behavioral Sciences*. *Behavioral Sciences* seeks to publish original research and scholarship contributing to our understanding of human behavior. The journal will provide a forum for work that furthers knowledge and stimulates research in the behavioral sciences. We are committed to building a diverse and methodologically rigorous literature of interest and benefit to behavioral and social scientists, as well as to clinical practitioners, educationalists, and the general public.

## Scope

In medical schools in the United States, teachers of the behavioral sciences are usually psychiatrists, and the content of teaching tends to concern general psychiatry alone. My own experiences were quite different in learning behavioral sciences in undergraduate psychology and in medical school. I recall that the curricula included topics such as group psychology, animal behavior, signal detection theory, learning theories, cultural anthropology, neurology and neurobiology, “happiness” research, doctor-patient communication, and cognitive psychology. This latter topic included processes that can distort thinking and lead to erroneous beliefs as exampled by studying the psychology of individuals that believed in psychic or paranormal phenomena. This was a wonderfully rich and stimulating education that fueled my interest in abnormal psychology and in psychiatry as a prospective career.

Such a diverse scope of the behavioral sciences is reflected by the interests and research activities of the 20 distinguished members of the Editorial Board. The 20 members have interests that include the neurobiology and genetics of behavior, the addictions, comparative psychology, cognitive psychology, animal models of behavior, learning, stress, epidemiology, and health psychology. The Board also has expertise ranging from child through adult psychiatry. This is an impressive list indeed but it is not complete. We hope to grow and internationalize our editorial board so please let us know of your research interests, expertise, and interests in serving.

## Research Methods

Such a diversity of scope of topics shares a commonality in the scientific methods deployed to advance knowledge. The methods are central to determining the validity of findings of research. In this regard, it is worth noting that the specific research question(s) drives the decision on study design. Thus, authors should explain their decision-making on how the design was chosen, and on the relative strengths and weaknesses of the specific methods (whether quantitative or qualitative in nature). The limitations of the methods are preferably reviewed in the discussion section so that the reader can judge the results, the potential for bias, and the adequacy of conclusions in light of the methods. We therefore ask our authors to be assiduous in detailing the methods.

## Editorial and Publication Processes and Peer Review

The editorial process will be led by our colleagues at MDPI. They will distribute manuscripts for peer review and consult the relevant editors regarding the results of the reviews. We wish to monitor our editorial processes and to respond in a very timely fashion to editorial queries, to completing reviews, and to editorial decision-making.

Submitted manuscripts will be sent to two or three reviewers for comments pertaining to the importance of the manuscript, quality of the methods, and adequacy of results and conclusions. We will also solicit comments on the adequacy of the references, utility of tables and figures, adequacy of the structured abstract and clarity of writing. The review process is critical to the scientific integrity of the discipline; reviewers should treat this responsibility with the upmost respect and empathy for authors. The editorial staff at MDPI and I hope to provide some information on how we are faring in our review and editorial decision-making processes in due course.

We are tremendously fortunate to have available the publishing experiences of the staff at MDPI. They will work to ensure timely reviews and editorial decision-making. *Behavioral Sciences* is strictly open access. This means that no reader will be charged for access to research published in the journal. The costs are covered through modest fees which are consistent with those charged by other open access journals. These are paid only if the article is accepted. In addition, fees for the inaugural volume will be waived. Open access publishing prevents considerable publishing delays and the high institutional subscription prices that are common to scientific publishing.

## Conclusion

We are all very excited about the adventures ahead for *Behavioral Sciences*. We will announce the theme and editors for the first issue very soon. We welcome additional proposals for guest editorships of theme issues. We especially welcome your submissions as original papers, scholarly or theoretical contributions, or focused and systematically conducted reviews. We also want to hear from you about how we can develop and improve our content and editorial decision-making processes, and please let us know of any interest in reviewing for us or in serving on the editorial board. This is your journal dear prospective readers, contributors and colleagues. On behalf of the Editorial Board, I sincerely thank you in advance for all of your efforts in supporting *Behavioral Sciences*!

